# Verticality Perceptions Associate with Postural Control and Functionality in Stroke Patients

**DOI:** 10.1371/journal.pone.0150754

**Published:** 2016-03-08

**Authors:** Jussara A. O. Baggio, Suleimy S. C. Mazin, Frederico F. Alessio-Alves, Camila G. C. Barros, Antonio A. O. Carneiro, João P. Leite, Octavio M. Pontes-Neto, Taiza E. G. Santos-Pontelli

**Affiliations:** 1 Department of Neuroscience and Behavior Sciences, Ribeirao Preto Medical School, University of Sao Paulo, Ribeirão Preto, São Paulo, Brazil; 2 Department of Ophthalmology, Otorhinolaryngology and Head and Neck Surgery, Ribeirao Preto Medical School, University of Sao Paulo, Ribeirão Preto, São Paulo, Brazil; 3 Department of Physics and Mathematics, Ribeirao Preto School of Philosophy, Sciences and Letters, University of Sao Paulo, Ribeirão Preto, São Paulo, Brazil; Charité University Medicine Berlin, GERMANY

## Abstract

Deficits of postural control and perceptions of verticality are disabling problems observed in stroke patients that have been recently correlated to each other. However, there is no evidence in the literature confirming this relationship with quantitative posturography analysis. Therefore, the objectives of the present study were to analyze the relationship between Subjective Postural Vertical (SPV) and Haptic Vertical (HV) with posturography and functionality in stroke patients. We included 45 stroke patients. The study protocol was composed by clinical interview, evaluation of SPV and HV in roll and pitch planes and posturography. Posturography was measured in the sitting and standing positions under the conditions: eyes open, stable surface (EOSS); eyes closed, stable surface (ECSS); eyes open, unstable surface (EOUS); and eyes closed, unstable surface (ECUS). The median PV in roll plane was 0.34° (-1.44° to 2.54°) and in pitch plane 0.36° (-2.72° to 2.45°). The median of HV in roll and pitch planes were -0.94° (-5.86° to 3.84°) and 3.56° (-0.68° to 8.36°), respectively. SPV in the roll plane was correlated with all posturagraphy parameters in sitting position in all conditions (r = 0.35 to 0.47; p < 0.006). There were moderate correlations with the verticality perceptions and all the functional scales. Linear regression model showed association between speed and SPV in the roll plane in the condition EOSS (R2 of 0.37; p = 0.005), in the condition ECSS (R2 of 0.13; p = 0.04) and in the condition EOUS (R2 of 0.22; p = 0.03). These results suggest that verticality perception is a relevant component of postural control and should be systematically evaluated, particularly in patients with abnormal postural control.

## Introduction

Postural control deficits are frequent and one of the most disabling problems in stroke survivors. These deficits alter the ability to maintain or change position [1] and are related to the lower quality of life [2] and longer time to recovery from stroke [3].

Several studies have demonstrated that stroke patients often exhibit altered vertical perception [4–8]. Altered Subjective Visual Vertical (SVV) is associated with weight bearing asymmetry in standing position and poor balance recovery [7,9]. Subjective Postural Vertical (SPV) and Haptic Vertical (HV) have also been shown to be correlated with postural deficits after stroke [4]. Surprisingly, the authors found a negative correlation between these three modalities of vertical perception and the Scale of Contraversive Pushing (SCP). This result is counter-intuitive, suggesting that impaired vertical perception is associated with better SCP performance.

We hypothesized that vertical perception had a positive correlation with postural control. To test this hypothesis, we analyzed the relationship between SPV and HV with quantitative posturography following stroke. We also assessed the relationship between these vertical perceptions and functional capacity.

## Materials and Methods

We evaluated patients with stroke (ischemic or hemorrhagic) consecutively admitted to our Emergency Unit. Patients were excluded if they were younger than 18 years or if they had at least one of the following: previous stroke with a modified Rankin scale (mRs) score greater than 1; another significant neurologic condition other than stroke; altered level of consciousness; cognitive deficit; receptive aphasia; severe concomitant systemic illness; visual deficit without corrective lenses; and orthopedic conditions that could interfere the postural balance. Cognitive screening was conducted using a validated Portuguese version of the Mini-mental state examination (MMSE) using cut-offs previously validated based on literacy status, 20 points for illiterate patients, 25 for 1 to 4 years, 26.5 for 5 to 8 years, 28 for 9 to 11 years and 29 for more than 11 years of education [10].

The ethics committee of University of Sao Paulo approved this study and written informed consent was obtained from all of the subjects.

Patients included in the study were evaluated after hospital discharge. Protocol assessments consisted of demographic and clinical data plus: National Institutes of Health Stoke Scale (NIHSS), which is composed of 11 items and the maximum score is 42 [11–13], the Modified Rankin Scale (mRs), which is used for measuring the degree of disability and varied from 0 to 6 [13], the Barthel Index (BI) that measures the patient’s performance in 10 activities of daily life and the maximum score is 100 [13,14], the Functional Independence Measure (FIM), which scores range from 18 (lowest) to 126 (highest) indicating level of function [15], the Ability for Basic Movement Scale (ABMS) that measures patient’s ability to performance basic movements at bedside and the maximum score is 20 [16], the Scale of Contraversive Pushing (SCP), where the patient is scored as having contraversive pushing if all three criteria are present, reaching a total score of at least 1 point [5,17] and Edinburgh Questionnaire which assess manual laterality and range from +1 to -1 [18].

All patients were also investigated for the presence of spatial neglect, sensory disturbance using an esthesiometer and peripheral vestibular dysfunction as assessed by oculomotor function, the head shake test, and head thrust test. These three tests together have a sensitivity of 63.6% and a specificity of 85.4% for detecting peripheral vestibular dysfunction [19].

### Vertical Perception Assessment

SPV and HV were evaluated in roll and pitch planes. All volunteers remained seated with eyes closed and practiced at least six trials to guarantee their understanding of the procedure.

**Fig 1:**
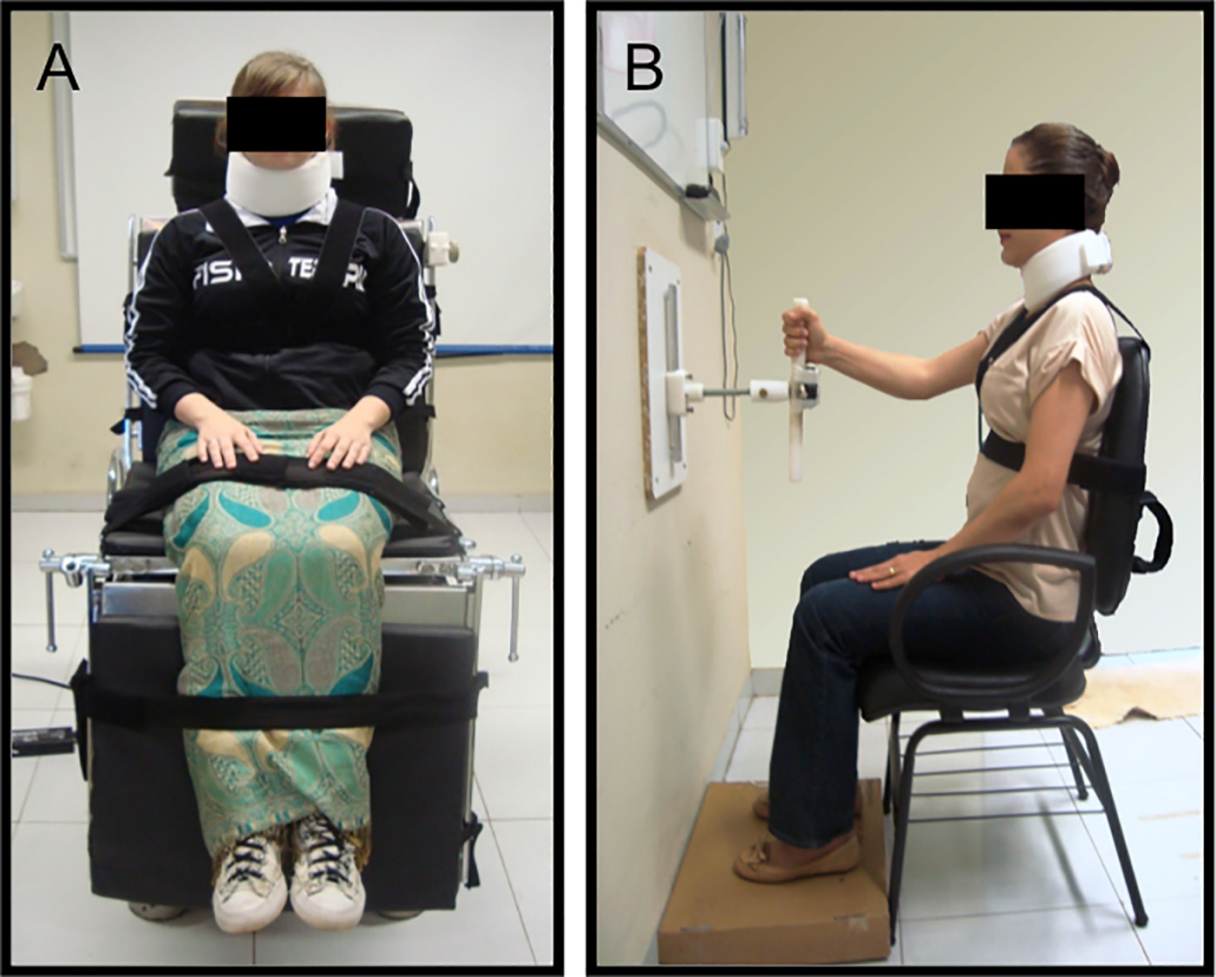
Position of the patient during SPV and HV tests. (A) SPV test. (B) HV test.

For SPV, the subjects were strapped in a sitting position to a mechanically tiltable chair, with trunk, thighs and legs restrained by bands and pads. We also restricted the head of the participants using a neck brace, and the feet remained unsupported (Fig 1A). The participants were randomly tilted away from true vertical at least 15° at a slow velocity, less than 1.5°/s, to avoid semicircular canal stimulation. After this, participants were told the direction the examiner had to move them to return to vertical. They were then instructed to tell the examiner to stop the chair rotation when they felt upright again. Before SPV evaluation, the equipment was calibrated using a goniometer and a pendulum.

For HV, we used a bar 40 cm in length and 4 cm in diameter fixed to the wall with its height above the floor adjusted to the patients' comfort. The examiner manually rotated the bar randomly to at least 15° off vertical in either direction. Patients were instructed to reset the bar to vertical with their unaffected hand (Fig 1B). SPV and HV inclinations were measured using a digital inclinometer with the precision of 0.01° with ten trials for each modality.

The individuals in this manuscript have given written informed consent (as outlined in PLOS consent form) to publish these case details.

The data of SPV and HV are described in degrees (°). In the roll plane, a positive sign indicated an ipsilesional tilt and a negative sign a contralesional tilt. In the pitch plane, a positive sign indicated a forward tilt and a negative sign a backward tilt.

### Balance Assessment

Posturography was performed using the 3D motion tracking by Polhemus® device. This device uses a magnetic source and sensors to track relative displacement. The magnetic source produces the electro-magnetic field and is the reference for the position and orientation measurements of the sensors. The sensors are devices whose position and orientation are measured relative to the magnetic source. The 3D displacement between the magnetic source and the sensors is registered by a board control. In this work, we placed the sensors on the skin of the patient and the magnetic source was fixed approximately 40 cm far from of the sensors. The patients were tested in sitting and standing positions. The experiment in standing position, two sensors were taped to the skin over the spinous process of the first thoracic vertebra and the sacral region and, in the sitting position, only one sensor was taped to the skin over the spinous process of the first thoracic vertebra.

In sitting position, patients sat on a wooden chair without back or feet support and upper limbs resting at their sides. In standing position, patients stood with their feet 15 centimeters apart with arms at their sides. They were asked to stand as quiet as possible and to look at a target 2 centimeters in diameter set at eye level 1 meter away. The unstable surface consisted in an Airex® Balance Pad over the wooden chair in the sitting position and on the floor in the standing position.

Data acquisition involved two trials of 90s [20] each under the following conditions: eyes open, stable surface (EOSS); eyes closed, stable surface (ECSS); eyes open, unstable surface (EOUS); and eyes closed, unstable surface (ECUS).

Posturography data were processed using the specially designed software written in LabView 8.0 (National Instruments). The acquired data was filtrated using a 4th order zero-lag low-pass Butterworth filter using the filtfilt function from Matlab (Mathworks—2011) with a cut-off frequency of 5 Hz and was adjusted for the height of each patient. The variables obtained in roll and pitch planes were: amplitude (cm), median displacement (cm), trajectory (cm), and speed (cm/s).

### Statistical Analysis

Statistical analyzes were performed using SPSS v17.0 and the level of statistical significance was set at 0.05 (two-sided). Descriptive statistics was used to assess clinical and demographic characteristics of all participants.

Spearman’s rank correlation coefficients were used to quantify the relationship between vertical perception and posturographic variables and with functional scales. The relationships between vertical perceptions and posturographic variables were assessed only within their respective planes (i.e. correlation between SPV and HV in the roll plane with posturographic speed in the roll plane). We also analyze the relationship between the planes (roll and pitch) among the variables: SVP, HV and posturographic variables.

A linear regression model was built to explain the relation between vertical perception and speed in sitting position. In this model, speed was considered as the dependent variable and the independent variables were the vertical perceptions, sensory deficit, age, and hemiparesis. We also built another linear regression model to verify the relation between vertical perceptions and functional scales (ABMS, FIM, and NIHSS).

## Results

We evaluated 355 stroke patients, of which, 310 were excluded. The main reasons for the exclusions were: 26.2% had another disease associated with stroke that influenced the assessment of vertical perception, 12.2% had altered level of consciousness, 15% previous stroke with an mRs score greater than 1, 16% died and 24% did not agree to participate in the study.

We included 45 stroke patients with a mean age of 64.4 ± 12.4 years, 60% were males and the mean years of study was 5.1 ± 3.4. The mean time from stroke to the first evaluation was 34.4 ± 9.9 days. The demographic and clinical characteristics of all participants are listed in Table 1.

**Table 1:** Demographic and clinical characteristics of stroke patients.

	Stroke patients (n = 45)
Age (years)	64.4 ± 12.4*
Gender–Males/Females	27 (60%)/18 (40%)
Years of study	5.1 ± 3.4*
BI	82.7 ± 20.9*
MMSE	23.8 ± 3.9*
FIM	106.6 ± 17.6*
SCP	0.9 ± 0.2*
NIHSS	1 (0–3)**
mRs	2 (1–4)**

BI: Barthel Index

MMSE: Mini-mental state examination

FIM: Functional Independence Measure

SCP: Scale for Contraversive Pushing

NIHSS: National Institutes of Health Stoke Scale

mRs: modified Rankin scale.

* data described as mean ±standard deviation

** data described as median and interquartil range.

The median SPV in the roll plane was 0.34° (interquartile range: -1.44° to 2.54°) and in the pitch plane was 0.36° (interquartile range: -2.72° to 2.45°). The median HV in the roll plane was -0.94° (interquartile range: -5.86° to 3.84°) and in the pitch plane was 3.56° (interquartile range:-0.68° to 8.36°).

Table 2 shows the results of posturographic assessments in sitting and upright positions. All patients completed the posturografic assessments in the sitting position, while in the standing position 28 patients completed the EOSS and ECSS conditions, 20 completed the EOUS condition and 16 completed the ECUS condition. In the sitting position, SPV in the roll plane was correlated with amplitude, trajectory and speed parameters as described in Table 3. SPV in roll plane was also correlated with trajectory and speed, in standing ECSS condition, with correlation coefficients of 0.38 (p = 0.02) and 0.35 (p = 0.04), respectively. We did not find correlations between SPV in the pitch plane and any posturographic parameter. For HV, we found a significant correlation between HV in roll plane and trajectory and speed in the standing ECSS condition, with correlation coefficients of 0.38 (p = 0.02) and 0.37 (p = 0.03), respectively.

**Table 2:** Posturographic assessment of stroke patients. Data described as mean ± standard deviation.

	Upright position				Sitting position			
Roll plane	EOSS	ECSS	EOUS	ECUS	EOSS	ECSS	EOUS	ECUS
Amplitude (cm)	2.2±1.07	4.71±2.1	2.90±1.56	21.5±40.5	1.33±1.21	1.84±1.29	2.19±4.17	1.78±1.73
Mean Displacement (cm)	-0.12±0.7	-0.34±1.46	0.08±1.02	0.63±1.99	0.04±0.49	0.03±0.69	-0.07±0.82	-0.1±0.8
Mean Trajectory (cm)	28.8±10.9	67.9±24.1	36.16±17.95	132.65±87.1	22.2±16.0	29.31±32.75	21.83±17.81	29.63± 30.91
Mean Speed (cm/s)	0.3±0.1	0.76±0.27	0.41±0.2	3.1±6.27	0.24± 0.17	0.33±0.36	0.25±0.21	0.33±0.34
**Pitch plane**								
Amplitude (cm)	2.5±0.8	4.4±1.54	3.34±1.5	7.15±3.05	2.61±3.2	2.99±2.61	2.76±2.87	2.75±2.23
Mean Displacement (cm)	-0.02±0.9	-0.16±0.8	0.08±1.21	-0.09±1.52	-0.38±1.12	-0.65±1.29	-0.35±1.64	-0.51±1.2
Mean Trajectory (cm)	39.9±15.8	78.37±31.5	51.71±31.56	124.07±53.18	37.4±28.02	44.25±47.19	33.11±25.68	48.96±54.78
Mean Speed (cm/s)	0.4±0.1	0.88±0.35	0.58±0.35	1.84±1.3	0.41±0.31	0.5±0.52	0.38±0.28	0.55±0.61

EOSS: eyes open stable surface

ECSS: eyes closed stable surface

EOUS: eyes open unstable surface

ECUS: eyes closed unstable surface.

**Table 3:** Correlation between SPV and posturographic parameters in roll plane at sitting position.

Posturographic parameters in roll plane	SPV in roll plane (°)							
	EOSS		ECSS		EOUS		ECUS	
	r	p	r	p	r	p	r	p
Amplitude (cm)	**0.34**	0.002**	**0.40**	0.006**	**0.47**	0.001**	**0.42**	0.004**
Mean Trajectory (cm)	**0.41**	0.006**	**0.50**	0.001**	**0.43**	0.003**	**0.47**	0.001**
Mean Speed (cm/s)	**0.41**	0.005**	**0.50**	0.001**	**0.44**	0.002**	**0.47**	0.001**

SPV: Subjective Postural Vertical

EOSS: eyes open stable surface

ECSS: eyes closed stable surface

EOUS: eyes open unstable surface

ECUS: eyes closed unstable surface.

**p < 0.012.

The analysis between planes showed no significant correlation between the SVP in roll plane versus in pitch plane (r = -0.79; p = 0.60). Similar result was found for correlation between HV in roll plane and pitch plane (r = 0.23; p = 0.11). The posturographic parameters: amplitude, mean displacement, trajectory and speed were also correlated between planes (roll versus pitch) and the results are described in Table 4.

**Table 4:** Correlation between posturographic parameters in roll and pitch planes for each condition in standing and sitting position.

	Amplitude (cm)		Mean Displacement (cm)		Trajectory (cm)		Speed (cm/s)	
	r	p	r	p	r	p	r	p
**Standing position**								
EOSS	0.61	0.0001*	0.19	0.28	0.68	0.0001*	0.67	0.0001*
EOUS	0.57	0.003*	0.21	0.29	0.65	0.0001*	0.65	0.0001*
ECSS	0.43	0.01*	0.01	0.92	0.77	0.0001*	0.78	0.0001*
ECUS	0.85	0.0001*	0.16	0.47	0.32	0.14	0.67	0.0001*
**Sitting position**								
EOSS	0.61	0.0001*	0.10	0.48	0.84	0.0001*	0.83	0.0001*
ECSS	0.42	0.003*	0.01	0.92	0.78	0.0001*	0.78	0.0001*
EOUS	0.57	0.0001*	0.11	0.45	0.86	0.0001*	0.87	0.0001*
ECUS	0.53	0.0001*	0.008	0.95	0.90	0.0001*	0.90	0.0001*

EOSS: eyes open stable surface

ECSS: eyes closed stable surface

EOUS: eyes open unstable surface

ECUS: eyes closed unstable surface.

*p < 0.05.

COP speed is described as the most reliable variable to measure balance in several population [20–24]. For this reason, we built a linear regression model between the average speed in sitting position and vertical perception. This showed an association between average speed and SPV in the roll plane in the EOSS condition (R2 of 0.37; p = 0.005), in the EOUS condition (R2 of 0.22; p = 0.03) and in the ECSS condition (R2 of 0.13; p = 0.04). We also found an association between average speed and age in the EOSS condition (R2 of 0.37; p = 0.007) and between average speed and hemiparesis in EOUS condition (R2 of 0.22; p = 0.03) and in the ECUS condition (R2 of 0.21; p = 0.03) (Table 5). We did not find any association between average speed and SPV in the pitch plane or HV in both planes (p > 0.05).

**Table 5:** Results from linear regression model between speed at sitting position and SPV in the roll plane.

Variable	Condition	Parameter	Parameter estimate	Standard error	t-statistics	R^2^	P-value
Speed Roll plane	EOSS	Intercepto	-0.125	0.126	-0.990	0.373	0.326
		Sensibilty	-0.071	0.054	-1.320	0.373	0.193
		Hemiparesis	0.106	0.058	1.830	0.373	0.075
		**Age**	**0.006**	**0.002**	**2.880**	**0.373**	**0.007**
		**SPV roll**	**0.055**	**0.019**	**2.980**	**0.373**	**0.005**
		HV roll	0.005	**0.004**	1.190	0.373	0.241
	EOUS	Intercepto	0.391	0.281	1.390	0.221	0.173
		Sensibilty	-0.160	0.120	-1.330	0.221	0.191
		**Hemiparesis**	**0.302**	**0.139**	**2.170**	**0.221**	**0.037**
		Age	-0.001	0.004	-0.190	0.221	0.853
		**SPV roll**	**0.095**	**0.043**	**2.210**	**0.221**	**0.033**
		HV roll	0.016	0.010	1.570	0.221	0.124
	ECSS	Intercepto	0.353	0.177	2.000	0.130	0.053
		Sensibilty	-0.048	0.074	-0.660	0.130	0.516
		Hemiparesis	-0.004	0.082	-0.050	0.130	0.963
		Age	-0.001	0.003	-0.520	0.130	0.609
		**SPV roll**	**0.056**	**0.027**	**2.110**	**0.130**	**0.041**
		HV roll	0.004	0.006	0.730	0.130	0.469
	ECUS	Intercepto	0.565	0.267	2.120	0.214	0.041
		Sensibilty	-0.207	0.114	-1.820	0.214	0.077
		**Hemiparesis**	**0.291**	**0.132**	**2.200**	**0.214**	**0.034**
		Age	-0.003	0.004	-0.770	0.214	0.446
		SPV roll	0.069	0.041	1.680	0.214	0.101
		HV roll	0.013	0.010	1.340	0.214	0.188

SPV: Subjective Postural Vertical

HV: haptic vertical

EOSS: eyes open stable surface

EOUS: eyes open unstable surface

ECSS: eyes closed stable surface

ECUS: eyes closed unstable surface.

Vertical perception and functional assessments were highly correlated, and the results are described in Table 6. We also made a linear regression model for these variables and we found an association between SPV in roll plane and ABMS (R2 of 0.33; p = 0.002) and FIM (R2 of 0.33; p = 0.008). The SPV in the pitch plane was also associated with ABMS (R2 of 0.16; p = 0.02). We did not find associations between HV in either plane and the functional assessments.

**Table 6:** Correlations between clinical and functional scales and SPV and HV in stroke patients.

	NIHSS	mRs	BI	FIM	ABMS	SCP
SPV roll plane	0.43 (0.001)**	0.18(0.22)	-0.34(0.01)**	-0.35 (0.01)**	-0.41 (0.003)**	0.35 (0.01)**
SPV pitch plane	0.42 (0.002) **	0.44 (0.001)**	-0.39 (0.004)**	-0.35 (0.01)**	-0.43 (0.001)**	0.33 (0.01)**
HV roll plane	0.32(0.02)*	0.27(0.05)*	-0.27(0.05)*	-0.29 (0.03)*	-0.33(0.01)**	0.20 (0.17)
HV pitch plane	0.28(0.04)*	0.24(0.11)	-0.29(0.03)*	-0.31 (0.02)*	-0.27(0.05)*	0.15 (0.31)

SPV: Subjective Postural Vertical

HV: Haptic Vertical

NIHSS: National Institutes of Health Stoke Scale

mRs: modified Rankin scale

BI: Barthel Index

FIM: Functional Independence Measure

ABMS: Ability for Basic Movement Scale

SCP: Scale for Contraversive Pushing.

* p < 0.05

**p < 0.012.

## Discussion

This is the first study to analyze the correlation of vertical perception with posturographic assessment in stroke patients. A prior study evaluated the impact of impaired vertical perception on body posture using the SCP. However, the authors found a negative correlation, indicating that patients with worse contraversive pushing behavior have more accurate vertical perception [4]. This result seems counter-intuitive. Our data show a consistent and positive correlation between SPV in roll plane and seated posturographic measures of amplitude, trajectory, and speed.

Our study is also the first to describe SPV in pitch plane in stroke patients. Previous authors have assessed this modality of vertical perception in healthy young people [25–27], elderly people [25,28] and subjects with vestibular disorders [29]. There is evidence of an association between SPV in the pitch plane and backward disequilibrium in elderly people [25]. However, we did not find this correlation in our sample of patients following stroke.

We believe that this is the first description of a significant correlation between SPV in roll plane and posturography following stroke. These results underscore the importance of accurate vertical perception for maintenance of postural control. Our study found more consistent findings in the roll plane rather than in the pitch plane. This may reflect lateralized weight bearing asymmetry away from the paretic side.

Previous studies reported a transmodal tilt in some stroke patients [4], as well as, intercorrelations between HV in roll and pitch planes [30,31]. In both cases, the authors suggest the involvement of a common neural circuit in the processing of spatial information in different planes and modalities. However, our study did not find correlation between planes in the SVP and HV, for this reason, more studies investigating SPV and HV in roll versus pitch plane in stroke patients are necessary to confirm this relationship.

Several brain areas have already been related to the postural control, as dorsolateral prefrontal cortices, supplementary motor area, right posterior parietal cortex, insula and basal ganglia [32–34]. Some of these areas were also connected to the construction of the internal model of verticality [4,35]. In our study we observed that postural changes in one plane can influence the postural control in another plane, but these relationship was not seen for vertical perceptions. These results indicate different brain areas for the processing of the spatial information related to postural control and vertical perceptions.

We found a significant correlation between HV in roll plane and posturographic parameters in standing position, but only for the ECSS condition. It is known that somatosensory information has a supplementary role in the construction of vertical perception [35]. Therefore, manual haptic vertical perception might not directly influence postural control.

Another important result is the correlation between vertical perception and the clinical and functional scales. A previous study has shown that abnormal SVV is associated with poor balance and low functional independence after a stroke [9]. However, our study is the first study to demonstrate a correlation between both SPV and HV and stroke severity (NIHSS) as well as functional level (mRs, FIM, ABMS, BI, and SCP). We also demonstrated a significant association between SPV in roll plane and the functional scales ABMS and FIM and an association between SPV in the pitch plane and ABMS. These results suggest a link between vertical perception, postural control and functionality following stroke.

It is important to note that interpretation of the present results might be constrained by the relatively small number of stroke patients included and their clinical characteristics. The hospital in which this study was conducted is a comprehensive stroke center that receives high complexity cases that did not meet our inclusion criteria. Although patients with mild functional deficits composed the majority of our sample, we found a significant and consistent correlation of vertical perception with posturographic assessment and functionality. Since stroke patients with mild deficits are usually able to walk and have a more active life, our results suggest that impaired vertical perception may be particularly important, as it may increase the risk of falls in this population.

Postural control is a highly complex task that integrates sensory and motor information to effect rapid postural adjustments. Additionally, other factors such as cognitive and musculoskeletal deficits influence the postural control after stroke and should be acknowledged [7,36–38]. Our results quantitatively confirm that SPV in roll plane is one more factor that can significantly influence postural control and functionality. For this reason, SPV should be systematically evaluated as a potential target for therapeutic strategies for postural control rehabilitation following stroke.

## Supporting Information

S1 File Complete Dataset. (XLSX)
